# Managing power dissipation in closed-loop reverse electrodialysis to maximise energy recovery during thermal-to-electric conversion

**DOI:** 10.1016/j.desal.2020.114711

**Published:** 2020-12-15

**Authors:** A.M. Hulme, C.J. Davey, A. Parker, L. Williams, S. Tyrrel, Y. Jiang, M. Pidou, E.J. McAdam

**Affiliations:** a Cranfield Water Science Institute, Cranfield University, Bedfordshire MK43 0AL, UK; b Centre for Creative and Competitive Design, Cranfield University, Bedfordshire MK43 0AL, UK; c Centre for Thermal Energy Systems and Materials, Cranfield University, Bedfordshire MK43 0AL, UK

**Keywords:** Distillation, Closed-loop, Recycle, Heat engine, Salinity gradient energy, Brine concentrate

## Abstract

Whilst the efficiency of reverse electrodialysis (RED) for thermal-to-electrical conversion has been theoretically demonstrated for low-grade waste heat, the specific configuration and salinity required to manage power generation has been less well described. This study demonstrates that operating RED by recycling feed solutions provides the most suitable configuration for energy recovery from a fixed solution volume, providing a minimum unitary cost for energy production. For a fixed membrane area, recycling feeds achieves energy efficiency seven times higher than single pass (conventional operation), and with an improved power density. However, ionic transport, water flux and concentration polarisation introduce complex temporal effects when concentrated brines are recirculated, that are not ordinarily encountered in single pass systems. Regeneration of the concentration gradient at around 80% energy dissipation was deemed most economically pragmatic, due to the increased resistance to mass transport beyond this threshold. However, this leads to significant exergy destruction that could be improved by interventions to better control ionic build up in the dilute feed. Further improvements to energy efficiency were fostered through optimising current density for each brine concentration independently. Whilst energy efficiency was greatest at lower brine concentrations, the work produced from a fixed volume of feed solution was greatest at higher saline concentrations. Since the thermal-to-electrical conversion proposed is governed by volumetric heat utilisation (distillation to reset the concentration gradient), higher brine concentrations are therefore recommended to improve total system efficiency. Importantly, this study provides new evidence for the configuration and boundary conditions required to realise RED as a practical solution for application to sources of low-grade waste heat in industry.

## Highlights

• Power dissipation using reverse electrodialysis in recycle is described.
• Recycle is more economically and energetically efficient than single-pass.
• Water flux, ionic transport and concentration polarisation impactful in recycle.
• Control of ionic concentration in dilute feed can improve system efficiency.
• Optimum current density is specific to chemical gradient.

## 1 Introduction

Approximately 20% of the world's population are without power, due to the absence of networked electricity, and the fragility of existing power grids, resulting in frequent large-scale power outages, particularly in low-income countries [[1], [2], [3]]. Conversely, waste heat energy is abundant, estimated to be equivalent to 246 PJ globally [4]. Thermal-to-electric conversion of this waste heat could provide a reliable source of off-grid power for small-scale applications. However, 63% of this energy source is classified as low-grade heat below 100 °C [4]. Conventional thermal-to-electric technologies, such as the Organic Rankine Cycle (ORC), are unsuitable for the conversion of low-grade heat as Carnot efficiency is directly proportional to the hot source temperature [5]. Furthermore, the specific cost per kW of small-scale ORCs in the range of 1–100 kW is prohibitively high [6]. Thermoelectric generators have recently been proposed for applications to transportation and manufacturing sectors, however widespread use is similarly hindered by high cost and low efficiency (<10%) [7].

**Fig. 1:**
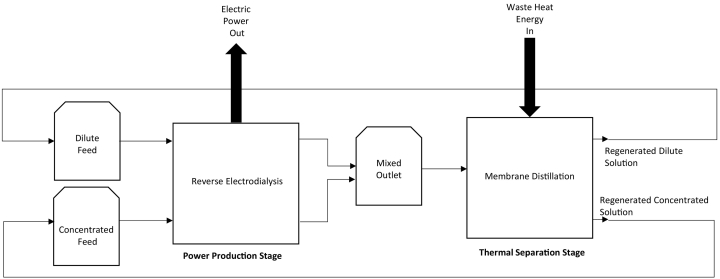
Diagram illustrating the principles of a reverse electrodialysis heat engine. Waste heat is utilised in the thermal separation stage to regenerate two solutions with differing salinities. These solutions are subsequently used to produce power by reverse electrodialysis.

In a reverse electrodialysis – membrane distillation heat engine, waste heat is utilised for the thermal separation of salt and water to produce two feeds with differing salinities [8]. The Gibbs free energy released by mixing solutions with a concentration gradient is then harnessed by reverse electrodialysis (RED) to produce power (Fig. 1). The proposed heat engine can theoretically achieve high energy efficiency of up to 85% [8]. Overall heat engine efficiency depends on both the thermal efficiency of solvent regeneration, and on maximising the Gibbs free energy which is recovered for power production. It has been theoretically demonstrated that membrane distillation (MD) can achieve exceptionally high ‘gain output ratios’, indicating substantial latent heat utilisation efficiency [9]. For small-scale applications of <1000 m^3^ day^−1^, MD outperforms alternative thermal separation stages such as multi-effect distillation in terms of energy consumption and gain output ratio [9]. Power production by RED has also been demonstrated to be scalable, from nano-scale and micro-fluidic applications [10] to large-scale (1 kW) pilot plants [11]. In an RED stack, anion and cation exchange membranes are alternately placed between two electrodes, creating concentrated and dilute compartments that initiate a concentration gradient driving ionic transport, where the separation of anions and cations is mediated by the membranes applied. An electrode rinse solution circulating at the electrode then converts ionic transport to an electric current [12]. Typically, RED applications have exploited naturally occurring feeds such as seawater and river water for power production, however, several critical differences must be considered when adapting RED for thermal-to-electric applications.

Initial RED research focussed on large-scale power production using seawater and river water. Following optimisation of stack design and operating conditions, power densities up to 2.2 W m^−2^ have been obtained using these feeds at ambient temperature [13]. The addition of a separation stage to restore the concentration gradient from the mixed RED effluent - enabling cyclic depletion and regeneration of the salinity gradient in a ‘closed-loop’ - has subsequently been investigated, with potential applications in energy storage and thermal-to-electric conversion. In the case of thermal-to-electric RED, the key distinction is that the working solution volume is fixed by the availability of heat energy to regenerate solution and restore the salinity gradient. The criticality is therefore to maximise the total power output from this fixed volume of solution prior to regeneration [14,15]. In contrast to conventional RED using naturally occurring saline feeds, the utilisation of pure artificial solutions in closed-loop RED minimises the likelihood of membrane fouling due to an absence of co-ions and impurities entering the system, in addition to enabling the selection of optimal fluid properties and operating conditions [8]. The available Gibbs free energy in a limited feed volume can therefore be increased by using a larger salinity gradient or alternative aqueous salt solutions. Consequently, a range of high solubility salts such as lithium bromide have been investigated for closed-loop RED and theoretically demonstrated to produce high overall system efficiency [8,16]. Regardless of the salt selected, power dissipation from a limited volume of working solution must be managed for closed-loop RED applications. In this work, sodium chloride is used as an example to determine how energy recovery from a fixed volume can be maximised. High power densities up to 6.7 W m^−2^ have been experimentally demonstrated using NaCl at high concentration and increased feed temperatures [17]. However, RED performance from a finite volume is a trade-off between power density and energy efficiency [18]. Energy efficiency is defined as the proportion of Gibbs free energy recovered for power production. Recycling feeds (ie reusing the feeds) increases the energy efficiency compared to typical single pass operation, without requiring a greater membrane area [14,17]. However, power densities decrease over time as exergy is lost from the system [14]. Management of exergy destruction is therefore paramount for overall system efficiency. Despite this, few studies have considered operating RED in recycle, or the effect of operating conditions and fluid properties on resulting exergy losses within the system.

Van Egmond et al. [14], investigated the effect of current density and temperature on the efficiency of an RED battery which is regenerated by electrodialysis. However, low efficiency in the electrodialysis charging stage (analogous to solution regeneration) limited the working domain to relatively low concentration gradients. In an RED heat engine, thermal separation by MD can be used to produce larger concentration gradients but it is unclear whether use of these feeds will benefit closed-loop performance. Daniilidis et al. [17], experimentally determined the energy efficiency and total work obtained by recirculating feeds across a range of concentrations, but neglected to consider the impact of operating conditions such as flow rate and current density. Giacalone et al. [19] modelled sources of exergy loss in RED, but did not examine the effect of feed temperature.

This work aims to determine how to manage discharge in closed-loop RED, by establishing boundary conditions which maximise the electrochemical potential harnessed for power production, whilst minimising exergy losses for high overall system efficiency in RED for thermal-to-electric applications. Specific objectives are to: (i) Benchmark the system in single pass, to characterise power density and open circuit voltage at maximum concentration gradient; (ii) Compare RED operation in single pass and recycle at different fluid velocities, to establish the trade-off between power density and energy efficiency, and identify for the first time the process conditions and preferred configuration to minimise exergy destruction and enable high energy efficiency; (iii) Examine power dissipation with a fixed volume in recycle, to identify the threshold for initiating thermal regeneration to maximise efficiency due to the limiting return on energy recovered; (iv) Determine the effect of sensible heat retention from thermal regeneration on the performance of RED in recycle.

## 2 Materials and methods

### 2.1 Experimental setup for reverse electrodialysis stack

A custom-made 5 cell-pair reverse electrodialysis stack was used. Neosepta AMX and CMX (Eurodia Industrie SA, Pertuis, France) ion exchange membranes with an active area of 10 cm × 10 cm were alternately stacked. Nylon woven spacers (SEFAR, Heiden, Switzerland) and silicon gaskets (Silex Silicones Ltd., Hampshire, UK) with a thickness of 0.3 mm separated the membranes. An extra cation exchange membrane sealed the electrode rinse compartment. Titanium mesh electrodes coated with a Ru/Ir mixed metal oxide with dimensions of 10 cm × 10 cm (MAGNETO special anodes, Schiedam, The Netherlands) were fixed inside each custom-made acetal endplate (Model Products LT, Bedfordshire, UK). The cell was bolted closed through the endplates. Peristaltic pumps fed the feed and electrode rinse solutions to the stack (Watson Marlow, Cornwall, UK). Feed temperature was controlled using refrigerated heating circulating baths (Model LT ecocool 150, Grant Instruments Cambridge Ltd., Cambridgeshire, UK). All tubes were insulated using nitrile rubber pipe insulation (Thick Kaiflex ST Class O, Pipelagging, Manchester UK). Four conductivity meters (2 CDH-SD1, Omega Engineering Limited, Manchester, UK and 2 Mettler Toledo Seven2Go Pro S7, Wolf Laboratories, York, UK) were fitted inline on the stack inlets and outlets. The feed reservoirs were each placed on a precision top loading balance (Model 4202E PT, Cole-Parmer Instrument Company, London, UK). This enabled a mass balance to be carried out and water transport within the stack to be quantified. Initial experiments were carried out in single pass to determine optimal operating conditions. Feed flow rates were varied from 5 ml min^−1^ to 200 ml min^−1^ using peristaltic pumps (Watson Marlow, Cornwall, UK). Feed temperature was increased from 10 °C to 50 °C to determine the effect on stack performance. To maximise energy efficiency, 250 g of each feed was continuously recycled through the stack.

### 2.2 Preparation of solutions

Aqueous sodium chloride solutions were prepared using 99% NaCl (Alfa-Aesar, Lancashire, UK) and deionised water. Solutions of concentration 0 M, 0.005 M, 0.01 M, 0.02 M, 0.04 M, 0.08 M, and 0.16 M were prepared for the dilute feed and 0.5 M, 1 M, 2 M, 3 M, 4 M, 5 M and a saturated solution prepared for the concentrated feed. A 1 l volume of electrode rinse was circulated through the stack at 200 ml min^−1^. The electrode rinse solution contained 0.1 M K_3_Fe(CN)_6_, 0.1 M K_4_Fe(CN)_6_ (Fisher Scientific, Leicestershire, UK) and NaCl (Alfa-Aesar, Lancashire, UK). A NaCl concentration halfway between the concentrated and dilute concentrations was used to minimise salt and water transport between the feeds and electrode rinse.

### 2.3 Electrochemical measurements

Electrochemical measurements were made using a potentiostat (IviumStat.h, Alvatek, UK) and data was logged using IviumSoft (IviumStat.h, Alvatek, UK). Prior to running a test, feed solutions were pumped through the stack until a stable (<0.01 V s^−1^) open circuit voltage (OCV) was obtained, indicating steady-state. All experiments were carried out at least three times, and reported as mean with error bars indicating standard deviation. In the single pass experiments, linear sweeps were carried out in galvanostatic mode, with current increased in steps of 2 × 10^−3^ A per second to determine the open circuit voltage (OCV) maximum current and maximum power density. The power density (P_d_) at time t is defined as the power produced per membrane area and can be calculated as follows:

(1) $$P_{d}=\frac{\mathrm{UI}}{\;A}$$

where I is the current (A), U is the voltage (V) produced by the stack and A is the total active membrane area (m^2^).

To determine energy efficiency, a constant current was drawn until no more work was produced at 0 V. The current was varied to determine its effect on energy efficiency at each salinity gradient. The total work which is produced by reverse electrodialysis can be determined:

(2) $$W_{\mathrm{RED}}=\sum_{t_{o}}^{t_{\mathrm{end}}}\mathrm{UI}∆t$$

where ∆t is the time interval (s), t_o_ is the time the current was started and t_end_ is the time at which no more work was produced [17].

The theoretical Gibbs free energy of mixing (ΔG_mix_) available if total mixing of the two solutions occurs is calculated using the Gibbs equation:

(3) $${\Delta G}_{\mathrm{mix}}={\Delta G}_{m}-\left({\Delta G}_{c}+{\Delta G}_{d}\right)$$

where ΔG is the Gibbs energy (J) with the subscripts m, c and d denoting the mixed outlet stream, the concentrate and dilute feeds respectively. Assuming the solutions are ideal:

(4) $${\Delta G}_{\mathrm{mix}}=-\left(N_{c}+N_{d}\right)T{∆S}_{m}-\left(-N_{c}T{∆S}_{c}-N_{d}T{∆S}_{d}\;\right)$$

where N is the number of moles (mol), T is the temperature (K) and ΔS is the Molar entropy (J K^−1^ mol^−1^) which can be calculated from:

(5) $$∆S=-R\sum_{i}x_{i}\ln\;x_{i}$$

where R is the universal gas constant, x is the mole fraction of species i. The energy efficiency (*η*_*RED*_) can then be determined as the ratio of the work produced (*W*_*RED*_) to the theoretical Gibbs energy [20]:

(6) $$\;\eta_{\mathrm{RED}}=\frac{W_{\mathrm{RED}}}{{\Delta G}_{\mathrm{mix}}}$$

## 3 Results and discussion

### 3.1 Large concentration gradients required for high power density in single pass

**Fig. 2:**
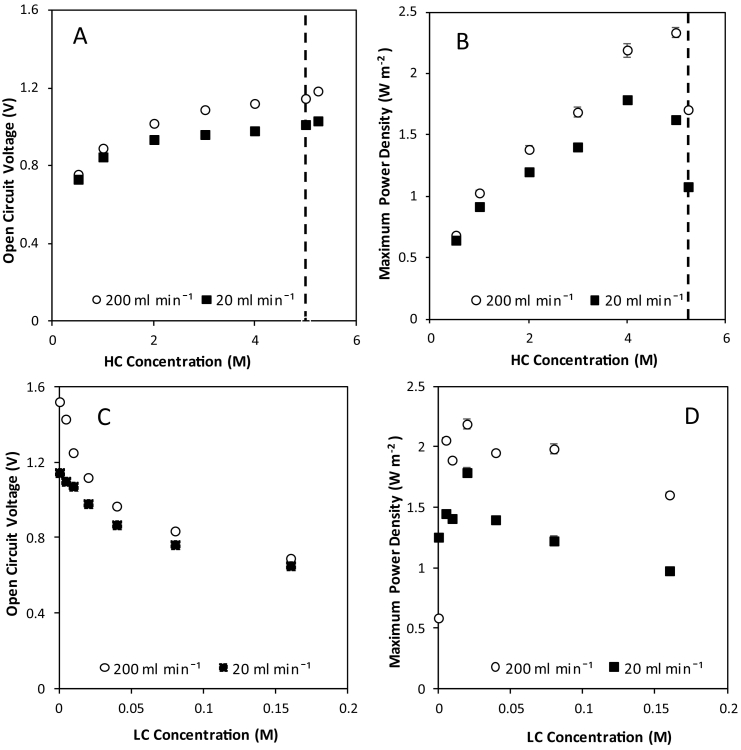
Effect of varying concentrated feed concentration with the dilute concentration fixed at 0.02 M on (A) open circuit voltage, and (B) maximum power density. Dilute feed concentration was then varied at a fixed 4 M concentrated feed on (C) open circuit voltage and (D) maximum power density. Galvanostatic sweeps were carried out in single pass (25 °C; Q_d_/Q_c_ = 1). Error bars represent the standard deviation of a triplicate. Dashed line in A and B shows the solubility limit of NaCl at the reference temperature.

To evidence the maximum achievable power density for the RED stack which comprised a fixed membrane area, evaluation was conducted in single pass (i.e. no reuse of feed), as this excludes the cumulative exergetic loss imposed by recycling the feed. Standard salt solutions with NaCl concentrations equivalent to sea and river water, 0.51 M and 0.02 M respectively, were initially used to benchmark with literature. Dilute and concentrated feeds were pumped to the cell at equal flow rates of 20 ml min^−1^. Experiments were then repeated at 200 ml min^−1^ to ensure flow rate did not limit performance due to the development of concentration polarisation. Open circuit voltage (OCV) is the voltage produced by the system at 0 A, and is a measure of the electrochemical potential difference across the RED stack. An OCV of 0.73 to 0.75 V was obtained for sea/river water feeds (Fig. 2A), which compares well to the theoretical OCV of 0.77 V. This demonstrates high membrane permselectivity of 94–97%, calculated from the ratio of the measured electrochemical potential to theoretical electrochemical potential [10]. Maximum power densities of 0.64 W m^−2^ at 20 ml min^−1^ and 0.67 W m^−2^ at 200 ml min^−1^ were obtained at the same conditions (Fig. 2B), falling in the range of 0.59–0.87 W m^−2^ expected from the literature for comparable systems using the same ion exchange membranes and electrodes at a range of compartment widths [13].

As the concentrated feed concentration was increased from 0.51 M to saturation at a fixed 0.02 M dilute feed, OCV increased up to 1.0 V at 20 ml min^−1^ and 1.2 V at 200 ml min^−1^ compared to a theoretical OCV of 1.4 V (Fig. 2A). Power densities approximately tripled to 1.79 W m^−2^ at 4 M (Fig. 2B). Large concentration gradients expectedly lead to improved voltages and power densities, due to the corresponding increase in Gibbs free energy available [17,21]. However, power density decreased as the concentration approached saturation. Similarly, Zhu et al. [21] established a peak in power density at 3.6 M. Conversely, Daniilidis et al. [17] reported the highest power density in the literature using 5 M and 0.01 M feeds at high temperature. This difference can be attributed to membrane ion exchange capacity [21]. Increasing the flow rate to 200 ml min^−1^ increased power density by 23% to 2.33 W m^−2^, due to the reduction in concentration polarisation at high flow rates [22]. As the dilute feed concentration was increased from 0 M–0.15 M at a fixed 4 M concentrated feed, OCV halved from 1.5 V to 0.65 V at 200 ml min^−1^ (Fig. 2C). This dramatic reduction in OCV following a small change in concentration demonstrates the increased sensitivity of the OCV to the dilute feed concentration in comparison to the concentrated feed, and highlights the importance of maintaining a low dilute feed concentration. Maximum power densities peaked at a dilute feed concentration of 0.02 M and decreased with further increases to the dilute concentration. Membrane, solution and spacer resistances dominate at lower dilute feed concentration, however these resistances decrease as the concentration of salt and hence ion conductivity is increased [17,21]. Above an optimal concentration, the benefit of reduced resistances is outweighed by the reduction in salinity gradient which limits power density [21]. Previous studies have similarly identified an optimal concentration of 0.005–0.02 M for the dilute feed [10]. The concentration gradient is the most significant factor governing achievable power density in single pass; increased power being achieved when complemented with an increased flow rate to reduce stack resistance and concentration polarisation effects.

### 3.2 Recycling feeds maximises energy efficiency from a fixed volume

**Fig. 3:**
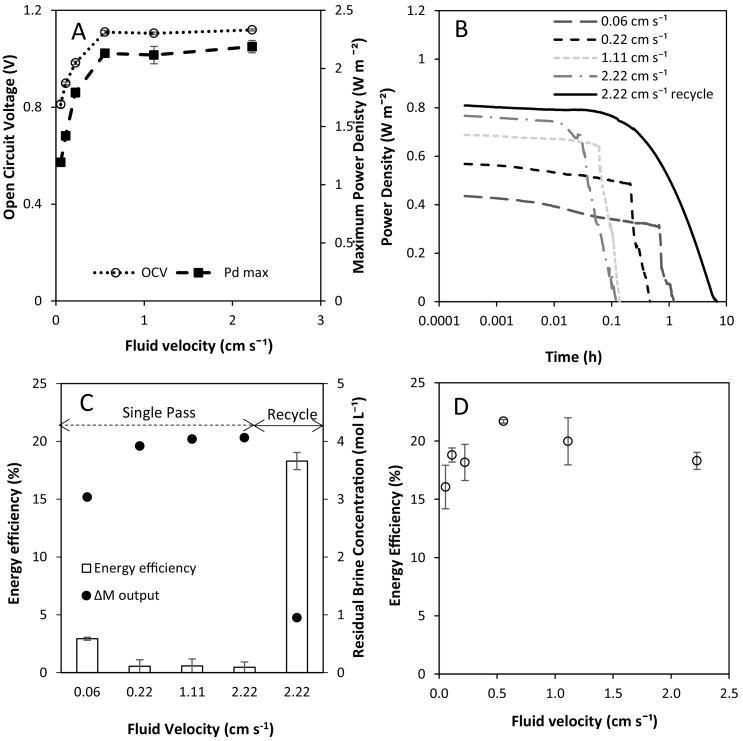
(A) Effect of fluid velocity in single pass on open circuit voltage and maximum power density with an excess of 4 M and 0.02 M feeds in single pass at 25 °C. (B) Power density over time from a fixed feed volume in single pass and recycle. (C) Energy efficiency and outlet concentration gradient from a fixed feed volume in single pass and recycle. (D) Effect of fluid velocity on energy efficiency from 250 g of 4 M and 0.02 M at 25 °C in recycle.

In single pass operation, increasing the flow rate of both feeds simultaneously from 0.06 cm s^−1^ to 0.55 cm s^−1^ caused OCV to increase from 0.8 V to 1.1 V, approaching the theoretical OCV of 1.3 V (Fig. 3A). Power density also increased up to a maximum of 2.1 W m^−2^ at 0.55 cm s^−1^ due to reduced concentration polarisation at increased flow rates [23,24]. The reduction in residence time is also likely to reduce exergy loss due to water and salt flux, further improving power density. Further increases to fluid velocity above 0.55 cm s^−1^ provided no further benefit to power density or OCV, as concentration polarisation is minimised. Similarly, Tedesco et al. [25] determined an optimal velocity of 1 cm s^−1^ for concentrated brines (5 M and 0.5 M), above which net power density reduces significantly.

Power density is initially constant in single pass when the feed volume in fixed, and is subsequently followed by a dissipation in power output, corresponding to the exhaustion of the available feed. Therefore, increasing the residence time through a reduction in velocity increases the duration of constant power production, but at significantly reduced power densities (Fig. 3B). For example, energy efficiency improved from 0.5 to 3% as fluid velocity was decreased from 2.2 cm s^−1^ to 0.06 cm s^−1^ (Fig. 3C). However, a significant residual concentration gradient of 3 M remained after a single pass. RED heat engine efficiency is dependent on both the energy recovered for power production by RED, and the efficiency of thermal regeneration. A residual concentration gradient between the streams exiting the RED stack represents unused electrochemical potential. Therefore, remixing these solutions prior to thermal regeneration represents an exergetic destruction, and maximum utilisation of the concentration gradient prior to thermal regeneration is critical for overall system efficiency.

By recycling the feeds continuously, a similar maximum power density to single pass at an equivalent velocity was achieved (P_d_, 0.8 W m^−2^; 2.2 cm s^−1^), and sustained for an order of magnitude longer, before a slow decline in power density is observed. This latter region enabled further energy recovery, but at a lower specific power output. The residual salinity gradient was reduced to <1 M, enabling energy efficiency of 18% to be obtained (Fig. 3C). This demonstrates that for a stack of equivalent membrane surface area, exergy destruction is best managed under recycle. In single pass, maximum energy efficiency is obtained at the lowest flow rate, as this extends residence time, which improves the utilisation of the concentration gradient. However, this reduction in velocity increases concentration polarisation and lowers power density. To illustrate, a power density of 0.4 W m^−2^ was obtained at 0.06 cm s^−1^, equating to 3% efficiency but half the power density achieved at 2.2 cm s^−1^. Whilst doubling the membrane area at 0.06 cm s^−1^ in single pass could deliver an equivalency in power output, this would increase the unitary energy cost (€ kWh^−1^) due to the capital investment. Furthermore, increased concentration polarisation at reduced flow rates would inevitably prevent energy efficiency equivalent to recycle from being obtained. Alternatively, equivalent energy efficiency to recycle could be achieved in single pass at an equivalent velocity (Fig. 3B, 2.2 cm s^−1^) through multiple stacks in series. Whilst delivering a higher absolute power output, this would also increase the unitary cost for energy production. Therefore, when scaling up RED for thermal-to-electrical applications, recycling feeds provides the most capitally efficient option for power production, through better governance over energy dissipation.

**Fig. 4:**
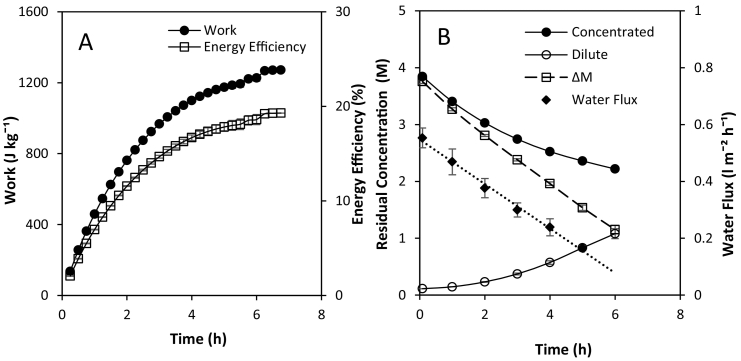
(A) Energy efficiency and work recovered over time, and (B) water flux and feed concentration profile over time from 250 g of 4 M and 250 g of 0.02 M in recycle. Feed flow rate, 200 ml min^−1^; feed temperature 25 °C; current 100 mA.

Varying the flow rate in recycle had little effect on performance; a plateau in maximum energy efficiency of 18–21% was obtained above a fluid velocity of 0.56 cm s^−1^ (Fig. 3D). We propose the relative insensitivity to flow rate in recycle, when compared to single pass (Fig. 2A) was due to increase ionic migration into the dilute feed which presents the primary resistance to power production. However, there is a temporal limit to be observed between sustaining power density above a set threshold, whilst seeking to minimise the unused electrochemical potential when operating in recycle (Fig. 4). Specifically, as the number of retention times increases, energy efficiency in recycle can be described in two stages: (i) a proportonal increase in work (J kg^−1^) with time whilst high power densities are sustained, followed by (ii) a subsequent rapid decline in work produced (Fig. 4A). Power densities decrease over time as exergy losses due to resistance, water transport and co-ion transport contribute to a dynamic concentration gradient (Fig. 4B) [14]. After approximately 4 h, the cumulative energy efficiency was observed to plateau. To maximise the return on investment in heat engine applications, it is therefore prudent to regenerate solutions to re-establish the salinity gradient at the intersection of these two regions, equivalent to around 80% of the maximum work available. However, the residual 2 M concentration gradient at this threshold represents substantial electrochemical potential, which in single pass could deliver a power density exceeding 1 W m^−2^ at the same concentration gradient (Fig. 2). This can be explained by the more complex boundary layer phenomenon which develops across channel length [24] over time in recycle. In contrast to single pass, where the dilute feed was fixed at 0.02 M, in recycle the concentration of the dilute feed has progressively increased to 1 M (Fig. 4B). At this concentration, we can infer that concentration polarisation has developed on the dilute and concentrated feed sides due to simultaneous ionic transport and water flux, and the dynamic temporal shift in these phenomena, with the attributable resistance inhibiting power production. Therefore, management of the dilute concentration is critical for energy efficiency in RED for thermal-to-electric conversion.

### 3.3 Large salinity gradients improve total work produced from a fixed volume at optimised current density

**Fig. 5:**
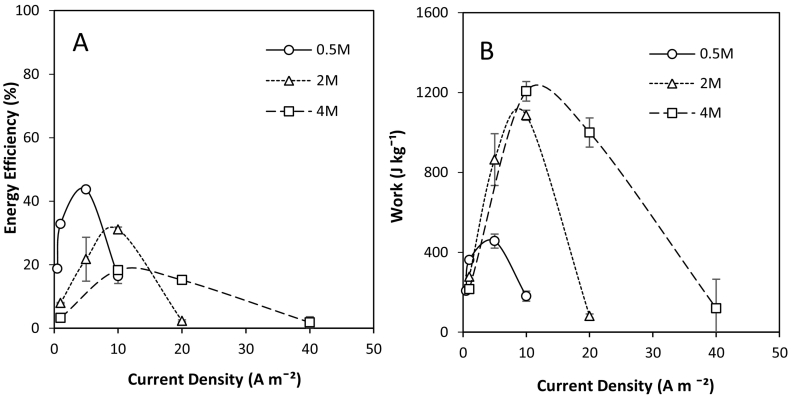
Effect of feed concentration and current density on: (A) energy efficiency; and (B) total work obtained from dilute and concentrated feeds in recycle. Feed flow rate, 200 ml min^−1^; feed temperature, 25 °C; solution mass, 250 g; dilute concentration, 0.02 M.

**Fig. 6:**
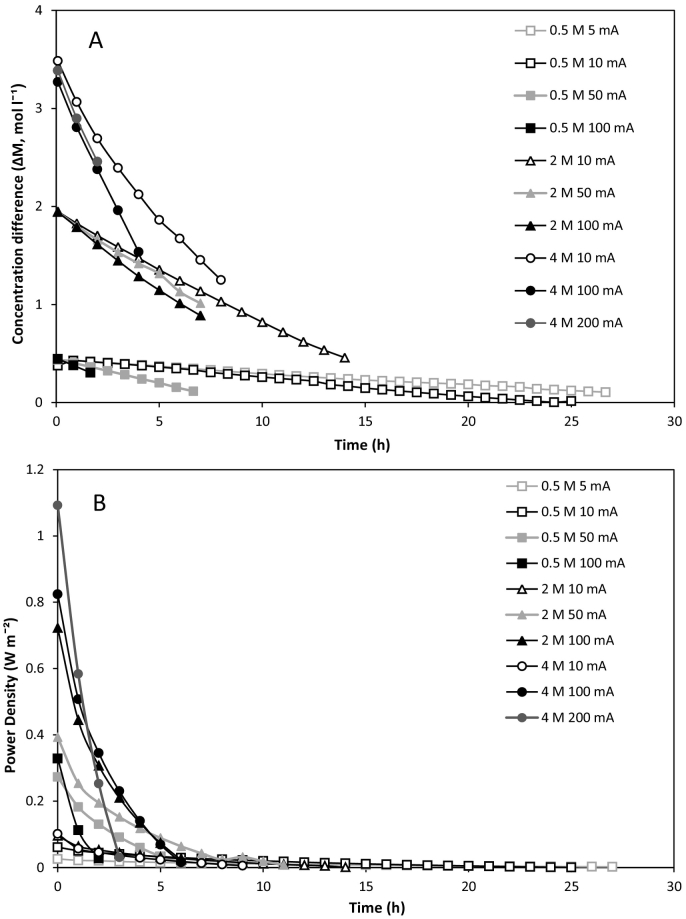
Effect of feed concentration and current density on: (A) change in concentration gradient over time; and (B) power density over time in recycle. Feed flow rate, 200 ml min^−1^; feed temperature, 25 °C; solution mass, 250 g; dilute concentration, 0.02 M.

For an RED-MD heat engine, the availability of waste heat determines the total feed volume which can be regenerated. Therefore, the work produced per volume of feed solution is an important performance parameter for closed-loop RED. The available Gibbs free energy in the fixed volume can be increased by using a larger salinity gradient. However, increasing the concentration gradient also increases the driving force for exergy losses from salt and water transport [19]. It must therefore be determined whether the increased potential energy established in the system using larger salinity gradients results in greater total work produced. At a 0.5 M concentrated feed concentration, equivalent to seawater, an energy efficiency of 43% was obtained at an optimised current density of 5 A m^−2^ (Fig. 5A). For comparison, at the equivalent current density (5 A m^−2^), the energy efficiency at 2 M concentrated feed concentration was 21%. Daniilidis et al. [17] similarly determined that the highest energy efficiencies were obtained at low concentration gradients. The same author reported a maximum total recovered energy of approximately 300 J kgˉ^1^ of feed solution at 2.5 M concentrated feed; recovered energy then decreased as the concentrated feed concentration was increased further. However this was carried out at a fixed current density, whereas, an enhancement in energy efficiency was observed in this study, through identification of optimal current density, which increased as the salinity gradient increased (Fig. 5A), the final maximum energy efficiencies being equal to 31 and 18% respectively for 2 M and 4 M. Whilst maximum energy efficiencies were below those identified at 0.5 M (work 456 J kg^−1^), increasing the concentration gradient vastly increased the total work produced from a fixed volume, to 1086 and 1206 J kg^−1^ for 2 M and 4 M respectively (Fig. 5B), and facilitated higher power densities (Fig. 6). However, above this optimum current density, the work produced per volume vastly reduces, which can be accounted for by the limited time available for power dissipation (Fig. 6). As such, despite the lower overall efficiency, large concentration gradients at optimised current densities are preferable for high power applications, due to the increased work available from the fixed solution volume. The difference in work provided by an increase from 2 to 4 M was not linear, which should be considered in the integrated RED-MD system, since the higher salinity gradient will reduce the vapour pressure, and therefore impact upon the thermal efficiency of MD, unless consideration has been given in the design.

### 3.4 Heating the feed doubles power density in single pass but reduces total work in recycle

**Fig. 7:**
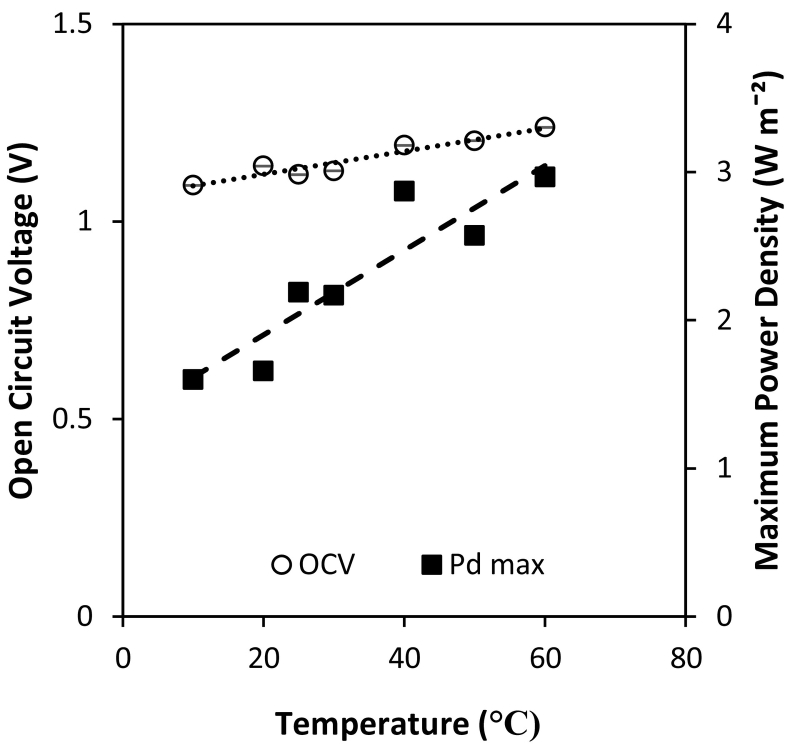
Effect of feed temperature on open circuit voltage and maximum power density in single pass. Concentrate and feed solution concentrations, 4 M and 0.02 M respectively; temperature, 25 °C; flow rate, 200 ml min^−1^.

**Fig. 8:**
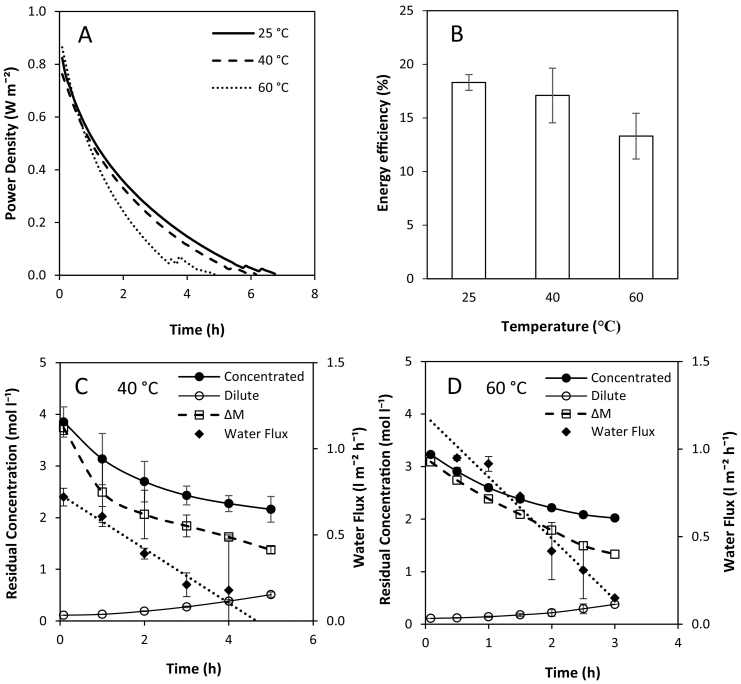
Effect of temperature on: (A) power density over time; and (B) energy efficiency obtained in recycle. Concentrate and feed solution concentrations, 4 M and 0.02 M respectively; solution mass, 250 g; flow rate, 200 ml min^−1^. Concentration profile and water flux at feed temperature of: (C) 40 °C; and (D) 60 °C.

**Fig. 9:**
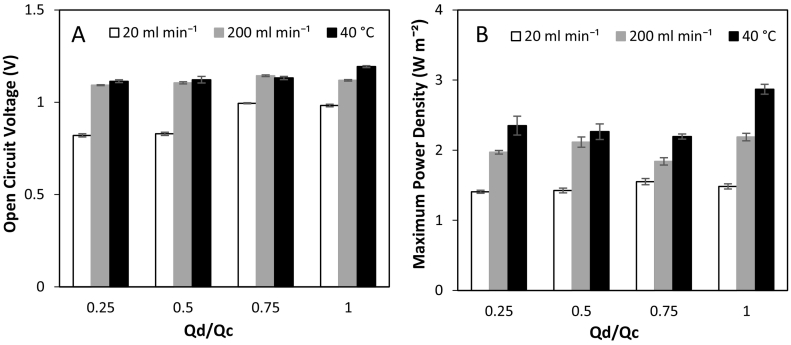
Effect of differing feed flow rates on: (A) open circuit voltage; and (B) maximum power density in single pass. Concentrate and feed solution concentrations, 4 M and 0.02 M respectively; concentrate flow rate was initially fixed at 20 ml min^−1^ and the dilute flow rate varied to produce flow ratios Q_d_/Q_c_ at 25 °C_._ Experiments repeated at a concentrate flow rate of 200 ml min^−1^ at 25 °C and 40 °C.

The retention of sensible heat from thermal regeneration presents an opportunity to increase RED feed temperatures at no additional cost, enabling increased power densities at a reduced membrane area and hence reduced capital investment. The impact of feed temperature from 10 to 60 °C was first tested in single pass, where a modest increase in OCV was observed from 1.1 V to 1.2 V, whilst power density almost doubled from 1.6 W m^−2^ to 3.0 W m^−2^ (Fig. 7). This is because membrane and solution resistances and permselectivity are reduced at increased temperatures [26]. A near linear increase in power density with temperature has previously been reported at high concentrations [17] and sea/river water concentrations [26]. Retention of sensible heat from thermal regeneration in RED could therefore represent a cost-effective method of producing higher power densities in thermal-to-electric applications; the sensible heat applied being potentially recoverable in spent solution to support the subsequent regeneration cycle. However, increasing feed temperature has the disadvantages of decreasing membrane permselectivity, and increasing ionic shortcuts, the spacer shadow effect and concentration polarisation [17,22]. Therefore despite the positive effect on power density in single pass, the cumulative effect of these phenomena in recycle may adversely affect power dissipation in recycle. This was evidenced by the decrease in time for power production as the temperature was increased (Fig. 8A). Energy efficiency decreased from 18% to 13% as the feed temperature was increased from 25 °C to 60 °C (Fig. 8B). This is because of the increase in water flux which causes the concentration gradient to decrease more rapidly at higher temperatures. At 40 °C, the concentration gradient was reduced to 1.5 M after 5 h (Fig. 8C). At 60 °C, the depletion in concentration gradient was significantly faster due to the increased water flux, which reached a concentration gradient of 1.3 M after only 3 h (Fig. 8D). As heating the feeds had a detrimental impact on RED performance, it is therefore proposed that heat recovery should be fully managed within the thermal separation stage, independent of RED. By utilising all available heat energy in the regeneration stage, instead of diverting heat to increase feed temperature in the power production stage, the total volume recovered by thermal regeneration could be improved. The improvement in efficiency of RED, coupled with the increased working volume is expected to improve the overall energy efficiency of the heat engine (Fig. 9).

## 4 Conclusions

In this study, recirculating RED feeds in a recycle mode has been demonstrated to provide the most suitable configuration for energy recovery from a fixed solution volume, providing a minimum unitary cost for energy production. This is significant as both unit cost and energy efficiency are acknowledged barriers to the viability of thermal-to-electric conversion for low-grade waste heat. Whilst application of RED to a sea water/river water matrix has been widely reported, high concentration gradients and operation in recycle present different challenges to optimisation. In single pass, power densities up to 3 W m^−2^ can be achieved through high concentration gradients and elevated temperatures (60 °C). However, when feeds are recycled, comparable conditions can promote water transport and concentration polarisation phenomena, which exert an exergy destruction that must be managed. Evaluation of power dissipation evidenced a two-phase transition, in which 80% of the work was recoverable over half of the discharge cycle, which appears the most economically pragmatic operating point to initiate regeneration. However, this results in a significant exergetic loss due to the high residual concentration gradient. The dilute feed has been identified as the critical limitation to energy recovery within discharge, due to polarisation and water flux effects. To reduce the exergetic loss from the fixed solution before regeneration, a staged configuration could therefore be considered through application of the residual concentrated feed to a second RED cell with a separate dilute feed, or inclusion of a new dilute feed to the existing cell. Optimum current density is specific to concentrated feed concentration, and whilst improved efficiencies are exhibited for lower concentrations, the recovered work from an equivalent working volume is far more significant at higher concentrations, although this is not proportional to feed concentration. When evaluated as an integrated system, an intermediate concentrated feed concentration may therefore present an optimum between work produced from a fixed solution volume, and the energy required for volumetric regeneration due to the raised vapour pressure gradient. Optimising RED stack configuration and process conditions can improve energy efficiency further, however, the present work evidences RED in recycle as a viable strategy for power generation from concentrated brines, improving the potential for delivering cost effective thermal-to-electrical conversion for application to small-scale, low-grade waste heat solutions.

## Declaration of competing interest

The authors declare that they have no known competing financial interests or personal relationships that could have appeared to influence the work reported in this paper.

## Appendix A

The use of differing flow rates for the concentrated and dilute feeds has been proposed as a strategy to minimise pumping energy whilst maintaining performance. For sea/river RED, Zhu et al. [27] demonstrated that a higher flow rate is required for the dilute feed in comparison to the concentrated feed, due to the increased solution resistance. For larger salinity gradients, a higher flow rate may be required for the concentrated feed to overcome increased concentration polarisation. To examine this, the flow rate of the dilute was varied at a fixed concentrate flow rate in single pass, to produce ratios defined as Q_D_/Q_C_. At 25 °C and 200 ml minˉ^1^ similar power densities of approximately 1.5 W m^−2^ were obtained at all ratios (Fig. 8). This is because the rapid diffusion of ions from the concentrated to the dilute feed, which is increased at higher concentration gradients, reduces the solution resistance of the dilute feed and so dilute flow rate no longer limits RED performance. However increasing the concentrated feed flow rate from 20 ml min^−1^ to 200 ml min^−1^ significantly improved both OCV and power density at all ratios. This is because concentration polarisation on the concentrated feed was limiting power production. Increasing the temperature to 40 °C further improved OCV and maximum power density at all flow ratios with a maximum power density of 2.87 W m^−2^ obtained at equal feed flow rates.
